# From the Gut to the Brain: Transcriptomic Insights into Neonatal Meningitis *Escherichia coli* Across Diverse Host Niches

**DOI:** 10.3390/pathogens14050485

**Published:** 2025-05-15

**Authors:** Lekshmi K. Edison, Subhashinie Kariyawasam

**Affiliations:** Department of Comparative Diagnostics and Population Medicine, University of Florida, Gainesville, FL 32608, USA

**Keywords:** neonatal meningitis *Escherichia coli* (NMEC), transcriptomics, adherence, host adaptation, biofilm formation, blood–brain barrier, metabolic reprogramming

## Abstract

Neonatal Meningitis-causing *Escherichia coli* (NMEC) is the leading cause of neonatal meningitis and exhibits remarkable adaptability to diverse host environments. Understanding its transcriptional responses across different host niches is crucial for deciphering pathogenesis and identifying potential therapeutic targets. We performed a comparative transcriptomic analysis of NMEC RS218, the prototype strain of NMEC, under four distinct host-mimicking conditions: colonic fluid (CF), serum (S), human brain endothelial cells (HBECs), and cerebrospinal fluid (CSF). Differential gene expression analysis was conducted to assess metabolic shifts, virulence factor regulation, and niche-specific adaptation strategies, in which RS218 demonstrated niche-specific transcriptional reprogramming. In CF, genes associated with biofilm formation, motility, efflux pumps, and cell division regulation were upregulated, aiding gut colonization. The serum environment triggered the expression of siderophore-mediated iron acquisition, enterobactin biosynthesis, and heme utilization genes, facilitating immune evasion and bacterial persistence. In HBECs, NMEC upregulated genes linked to nucleoside metabolism, membrane remodeling, pilus organization, and blood–brain barrier (BBB) traversal. In CSF, genes related to oxidative stress resistance, chemotaxis, DNA repair, biofilm formation, and amino acid biosynthesis were enriched, reflecting NMEC’s adaptive mechanisms for survival under nutrient-depleted conditions. Energy-intensive pathways were consistently downregulated across all niches, highlighting the need for an energy conservation strategy. This study provides novel insights into NMEC’s adaptive strategies across different host environments, emphasizing its metabolic flexibility, virulence regulation, and immune evasion mechanisms, offering potential targets for therapeutic intervention.

## 1. Introduction

Neonatal meningitis, a life-threatening condition predominantly affecting preterm and low-birth-weight infants, continues to be a major global health concern [1]. Among its causative agents, Neonatal Meningitis *Escherichia coli* (NMEC), a pathotype of extraintestinal *E. coli*, is the most common Gram-negative pathogen associated with this disease [2]. NMEC infections lead to devastating outcomes, including long-term neurological damage and high mortality rates [3]. The NMEC’s ability to cross the blood–brain barrier (BBB) and establish infection within the central nervous system (CNS) defines its virulence and pathogenesis [2]. Despite advancements in neonatal care and antimicrobial therapy, neonatal meningitis due to *E. coli* remains an important medical issue in infants. However, our understanding of NMEC survival strategies and the molecular mechanisms of infection remains incomplete, hindering efforts to develop effective interventions.

NMEC encounter diverse and challenging host environments during infection, including nutrient-rich but hypoxic conditions in the colon, iron-restricted serum, nutrient-limited cerebrospinal fluid (CSF), and human brain endothelial cells (HBECs) that form the BBB [4,5]. Each environment imposes unique selective pressures that require NMEC to dynamically reprogram gene expression to adapt, survive, and proliferate. For example, the colon represents a highly competitive niche where NMEC must outcompete the resident microbiota for nutrients [6], while the serum and CSF present nutrient-scarce conditions coupled with host-imposed iron limitation [7]. Additionally, crossing the BBB to reach the CNS is a critical step in NMEC pathogenesis, which requires specialized mechanisms for adhesion, invasion, and immune evasion [8]. However, the molecular mechanisms enabling NMEC to adapt to these diverse niches remain poorly understood.

While previous research has provided valuable insights into NMEC’s virulence factors, such as the K1 capsule, S-fimbriae, and IbeA protein [9,10], significant gaps remain in our understanding of its niche-specific transcriptomic adaptations. Most studies have focused on either in vitro experiments [11] or broad descriptions of virulence-associated genes [10,12] without considering the unique pressures of physiologically relevant host environments. How NMECs adjust their metabolism, stress response systems, and virulence factor expression in response to specific host environments, such as colonic fluid (CF), serum, HBECs, and CSF, remains largely unexplored. For instance, which genes are activated to scavenge nutrients under iron-limited conditions? How does NMEC mitigate oxidative stress in the host? What specific mechanisms are employed to adhere to host tissues and cross critical barriers such as the BBB? These vital questions remain unanswered.

Another key research gap lies in understanding how NMEC’s metabolic reprogramming and virulence strategies interplay in different host environments. Emerging evidence suggests that pathogens fine-tune their gene expression to prioritize survival and colonization in each environment [13]; however, the extent of these adaptations in NMEC remains unclear. This lack of knowledge has hindered the development of niche-specific therapeutic targets and antimicrobial interventions. Advancements in high-throughput RNA sequencing (RNA-seq) have revolutionized our ability to study bacterial transcriptomes under host-relevant conditions. By leveraging this technology, our study aims to fill these critical knowledge gaps by comprehensively profiling the transcriptomic adaptations of the NMEC strain RS218 in four distinct host-relevant environments: CF, serum, HBECs, and CSF. By comparing NMEC gene expression in these environments to a standard bacterial culture medium, we sought to uncover key metabolic shifts, stress response mechanisms, and virulence strategies that enable NMEC to thrive and establish infection in the neonatal host.

## 2. Materials and Methods

### 2.1. Bacterial Strain

The NMEC strain RS218 (O18:H7:K1), originally isolated from the CSF of neonates with meningitis in the 1970s, was kindly provided by Dr. James Johnson from the University of Minnesota, St. Paul, MN, USA. For routine maintenance, the strain was cultured in Luria Bertani (LB) broth (BD Difco, Franklin Lakes, NJ, USA) at 37 °C with shaking at 200 rpm.

### 2.2. Cell Culture and Media

The HBEC-5i human cerebral microvascular endothelial cell line (ATCC CRL-3245), purchased from ATCC (Manassas, VA, USA), was cultured in T75 flasks coated with 1% gelatin (ATCC PCS-999-027) in DMEM/F-12 medium (ATCC 30-2006) supplemented with 10% fetal bovine serum (FBS; Atlanta Biologicals, Flowery Branch, GA, USA) and microvascular endothelial cell growth components (ATCC PCS-100-030). The cultures were incubated at 37 °C in a humidified incubator with 5% CO_2_ and passaged every 3–4 days after dissociating the cells with 0.05% trypsin-EDTA (Gibco, Brooklyn, NY, USA) upon reaching 80–90% confluency.

### 2.3. Incubation of NMEC Strain RS218 in Host-Stimulating Environments

**Figure 1:**
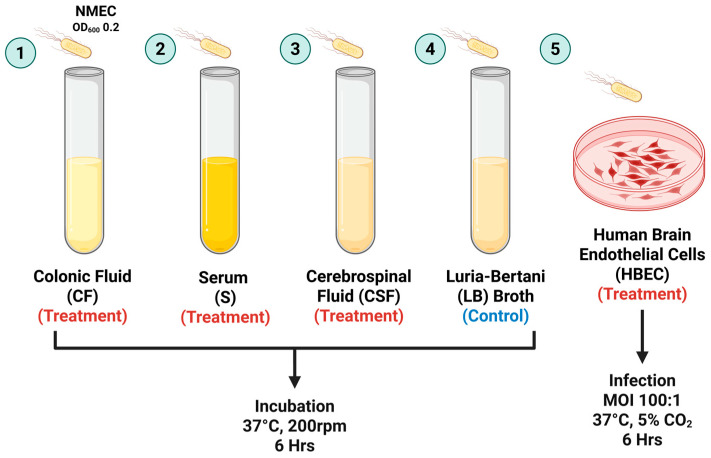
Experimental design illustrating neonatal meningitis *E. coli* (NMEC) RS218 exposure to host-mimicking environments and infection conditions.

As illustrated in Figure 1, to simulate host-specific niches encountered by NMEC RS218 during neonatal meningitis infection, the strain was incubated in various host-relevant environments, including CSF (Biochemazone Inc., Leduc, AB, Canada), CF (Biochemazone Inc., Leduc, AB, Canada), serum (Innovative Research, Novi, MI, USA), and HBECs. NMEC RS218 cultures were grown overnight in LB broth at 37 °C, shaking at 200 rpm. Bacterial cultures were harvested by centrifugation at 4000× *g* for 10 min at 4 °C and washed twice with sterile phosphate-buffered saline (PBS, pH 7.4). The bacterial pellet was resuspended in PBS, and the suspension was adjusted to an optical density (OD) of 0.2 at 600 nm. The bacterial suspension was incubated separately in the CSF, serum, and CF at 37 °C with shaking at 200 rpm for 6 h. LB broth was used as the control for baseline gene expression.

For the HBEC infection model, HBEC-5i cells were seeded at a density of 2.1 × 10^6^ cells per T75 flask (Corning Life Sciences, Tewksbury, MA, USA) pre-coated with 1% gelatin. Cells were grown in DMEM/F-12 medium supplemented with 10% fetal bovine serum (FBS; Atlanta Biologicals, Flowery Branch, GA, USA) and endothelial cell growth supplements. The cultures were maintained in a humidified incubator at 37 °C with 5% CO_2_ until confluent monolayers were obtained. Overnight-grown NMEC RS218 cultures were washed twice with PBS and resuspended in serum-free DMEM/F-12 to achieve an OD_600_ of 0.2. Prior to infection, HBECs were washed and maintained in serum-free DMEM/F12. Bacteria were added to HBEC monolayers at a multiplicity of infection (MOI) of 100:1. The co-culture was incubated at 37 °C in a 5% CO_2_ atmosphere for 6 h. After incubation, the unattached bacteria were removed by washing the monolayers thrice with sterile PBS. Following incubation, bacterial cultures from each growth condition (CSF, serum, CF, and HBECs) were pelleted by centrifugation at 10,000× *g* for 10 min at 4 °C and stored in RNA*later* (Invitrogen, Waltham, MA, USA) at −80 °C until RNA extraction and downstream transcriptomic analyses.

### 2.4. Total RNA Extraction, Microbial RNA Enrichment, and mRNA Purification

Total RNA was extracted using the RiboPure RNA Purification Kit (Invitrogen, Waltham, MA, USA) and further processed for rRNA depletion to enrich for bacterial mRNA using the MICROBExpress Bacterial mRNA Enrichment Kit (Invitrogen, Waltham, MA, USA). All steps were performed according to the manufacturer’s instructions. The quality and concentration of RNA samples at each step were measured using a Qubit™ 4.0 Fluorometer (Thermo Fisher Scientific, Wilmington, DE, USA).

### 2.5. Transcript Library Construction and Sequencing

RNA libraries were prepared using the TruSeq Stranded mRNA Library Kit (Illumina, San Diego, CA, USA). Enriched mRNA was fragmented and primed for the synthesis of first- and second-strand complementary DNA (cDNA). The resulting double-stranded cDNA fragments were adenylated at the 3′ end, ligated to multiple index adapters (TruSeq RNA Combinatorial Dual Indexes; Illumina Inc., San Diego, CA, USA), and enriched by PCR amplification. The cDNA libraries were multiplexed and clustered across two lanes of a flow cell. Sequencing was performed on the NovaSeq X Series platform (Illumina Inc.), generating 2 × 150 bp paired-end (PE) reads with an average output of 375 Gb per run.

### 2.6. Sequence Analysis

Raw sequencing reads generated from the NovaSeq X Series platform were analyzed using the CLC Genomics Workbench (Qiagen, Redwood City, CA, USA). The quality of the raw reads was assessed, and adapter sequences and low-quality bases (Phred score < 30) were trimmed. High-quality reads were subsequently mapped to the *E. coli* RS218 reference genome assembly ASM81734v1 (RefSeq assembly GCF_000817345.1) obtained from the National Center for Biotechnology Information (NCBI). Reads were aligned to the reference genome using default settings in the CLC Genomics Workbench. Gene expression was calculated using the RPKM (reads per kilobase of exon model per million mapped reads) and applying the equation: RPKM = number of gene reads/(mapped reads (millions) × gene length (kb)). False discovery rate (FDR) was used to identify statistically significant alterations in gene expression. To identify differential gene expression (DGE) between the two groups (test vs. control), TMM Normalization (Trimmed Mean of M values) described for Whole-Transcriptome RNA-seq Technology, was applied. Samples were analyzed in triplicate, and the results were averaged to obtain fold changes (FCs). Three gene filtering criteria were applied to the differentially expressed data: FDR ≤ 0.05, FC ≥ 2.0, and *p*-value ≤ 0.01. Gene enrichment analysis was performed using ShinyGO 0.77 and iDEP 1.1.

### 2.7. RNA Isolation, cDNA Synthesis, and Quantitative RT-PCR (qRT-PCR) Validation of RNA-seq Data

The RiboPure RNA Purification Kit (Invitrogen) was used to extract total RNA from the bacterial cultures in the treatment and control groups. One microgram of total RNA from each sample was used for first-strand cDNA synthesis using the iScript cDNA Synthesis Kit (Bio-Rad Laboratories Inc., Hercules, CA, USA) according to the manufacturer’s instructions. Primers were designed using Primer3Plus (https://www.primer3plus.com/index.html, accessed on 12 December 2024) and synthesized by Integrated DNA Technologies, Inc. (San Diego, CA, USA). Primer sequences for RT-qPCR analysis of the selected genes are listed in Supplementary File S1. The SsoAdvanced Universal SYBER^®^ Green Supermix (Bio-Rad Laboratories Inc.) was used for RT-qPCR analysis using a QuantStudio 5 Real-Time PCR instrument (Applied Biosystems, Carlsbad, CA, USA). A 10 µL volume of SsoAdvanced Universal SYBER Green Supermix, 2 µL cDNA template, 0.8 µL each of the forward and reverse primers, and 7.2 µL of sterile, nuclease-free water were added to the 20 µL PCR volumes. PCR was performed under the following conditions: 95 °C for 3 min and 40 cycles of 95 °C for 15 s and 60 °C for 30 s. The comparative CT approach (2^−ΔΔCT^) was used for the quantitative gene expression experiments. Expression data were normalized using 16S rRNA. Each qPCR reaction was performed in triplicate, and experiments were conducted in three independent biological replicates. The results are presented as means ± standard deviation (SD). Statistical significance was evaluated using unpaired two-tailed Student’s *t*-test in GraphPad Prism version 10.0.1, with *p* < 0.05 considered statistically significant. Correlation analysis between qPCR and RNA-seq fold changes was conducted using Pearson’s correlation coefficient (R^2^) to assess consistency between the datasets.

### 2.8. Statistical Analysis, Software, and Data Preparation

Statistical analysis of DGEs was performed with the CLC Genomics Workbench using a General Linear Model with a negative binomial distribution. Statistical significance was set at *p* < 0.05. Graphs were plotted using GraphPad Prism version 10.0.1 (GraphPad Software, Inc., San Diego, CA, USA). All quantitative analyses were performed in triplicate in independent experiments.

### 2.9. Data Availability

The transcriptomic profile data (raw and processed) described in this study were deposited in the Gene Expression Omnibus (GEO) database in NCBI under accession number GSE291265.

## 3. Results

### 3.1. Overall Transcriptome Profiling

**Figure 2:**
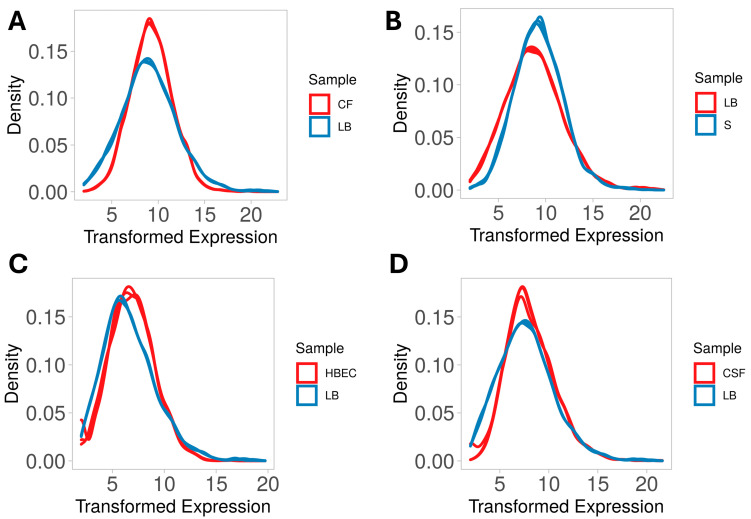
Density plots of transformed gene expression in neonatal meningitis *E. coli* (NMEC) RS218 across different host niches, such as colonic fluid (CF) (**A**), serum (S) (**B**), human brain endothelial cells (HBECs) (**C**), and cerebrospinal fluid (CSF) (**D**) illustrate differences in gene expression distribution, with shifts indicating transcriptional changes driven by niche-specific host factors.

In this study, we analyzed the expression of genes encoding stress response systems and niche-specific factors in NMEC strain RS218 grown in four host-simulating environments, namely, CSF, CF, serum, and HBECs, to identify the adaptive mechanisms employed by NMEC in each host-relevant niche. To determine DGE, bacteria were incubated in CSF, CF, and serum and co-cultured with HBECs for six hours. Total RNA extracted under these conditions had RNA Integrity Number (RIN) values ranging from 7.5 to 9.0. For serum and HBECs, RNA samples were enriched for bacterial RNA, and ribosomal RNA was depleted in all samples. The resulting bacterial mRNA was subjected to high-throughput sequencing, achieving an average coverage of 10 billion reads per sample. The transcript reads obtained were successfully mapped to the NMEC RS218 reference genome of *E. coli* RS218 (ASM81734v1). The mapping efficiencies of CF, serum, HBECs, and CSF samples ranged from 98.36% to 99.74%, respectively. For the LB broth control, the percentage of mapped reads was 97.82%. Diagnostic plots were generated to analyze variations in library sizes and read distributions across all treated samples versus controls, ensuring data quality and consistency (Figure 2A–D).

**Figure 3:**
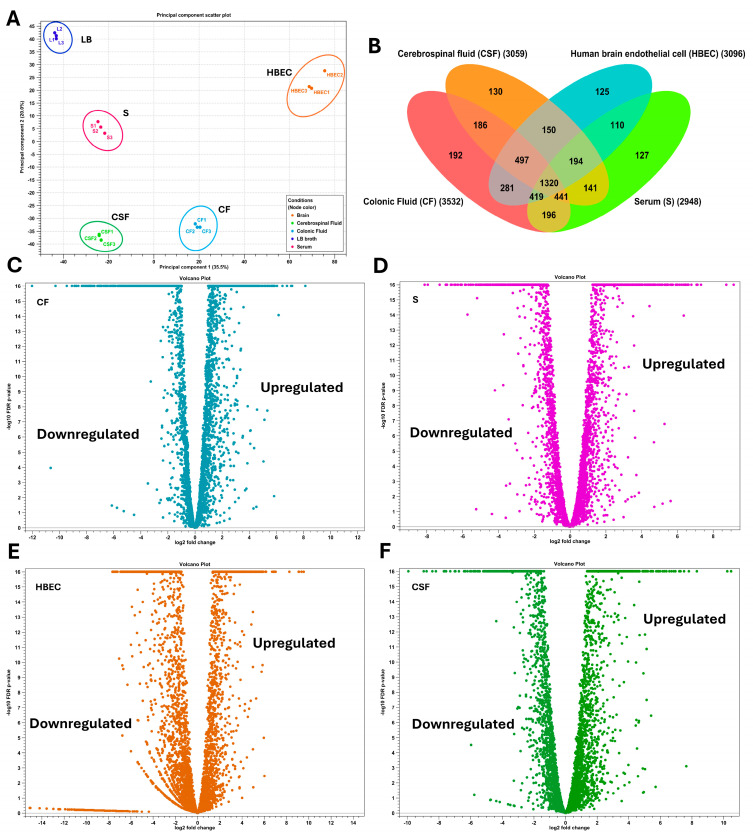
Principal component analysis (PCA), gene expression overlaps, and differential gene expression patterns across host niches. (**A**) The PCA plot illustrates a distinct clustering of samples, indicating environment-specific gene expression patterns. Colonic fluid (CF), serum (S), cerebrospinal fluid (CSF), human brain endothelial cells (HBECs), and control Luria–Bertani (LB) broth are color-coded, with replicates clustered closely together, reinforcing the reproducibility of transcriptomic shifts. (**B**) Venn diagram representing the distribution of the expressed genes under each condition. Volcano plots display the log_2_ fold-change in gene expression against the −log_10_ adjusted *p*-values (FDR ≤ 0.05) for CF (**C**), serum (**D**), HBEC (**E**), and CSF (**F**) host niches compared to LB. Each dot represents an individual gene, with significantly upregulated genes on the right and downregulated genes on the left.

Differential expression analysis was performed using the CLC Genomics Expression Browser tool, with filtered criteria including a two-fold change, false discovery rate (FDR) ≤ 0.05, and *p*-value ≤ 0.05. The transcriptomic profiles of NMEC RS218 were distinct across the host-stimulating environments compared to the LB control, as shown by the principal component analysis (PCA) plot (Figure 3A), which illustrated a clear separation between conditions, indicating niche-specific gene expression patterns. The total expressed genes in CF, serum, HBECs, and CSF were 3532, 2948, 3096, and 3059, respectively. Specifically, 192 genes were uniquely expressed in CF, 127 in serum, 125 in HBECs, and 130 in CSF. Additionally, 1320 genes were expressed under all conditions (Figure 3B). Supplementary File S2 includes each condition’s detailed, unfiltered differential gene expression profiles. In CF, 1558 genes were upregulated, and 1974 genes were downregulated compared to LB. For serum and HBECs, 1252 and 1306 genes were upregulated, respectively, while 1696 and 1790 were downregulated. In CSF, 1440 genes were upregulated, and 1619 were downregulated. Volcano plots illustrating differential expression patterns are provided in Figure 3C–F, and heat maps of all DEGs are depicted in Supplementary Figures S1–S4.

### 3.2. NMEC’s Transcriptomic Adaptations for Thriving in the Competitive and Hypoxic Colonic Environment

**Figure 4:**
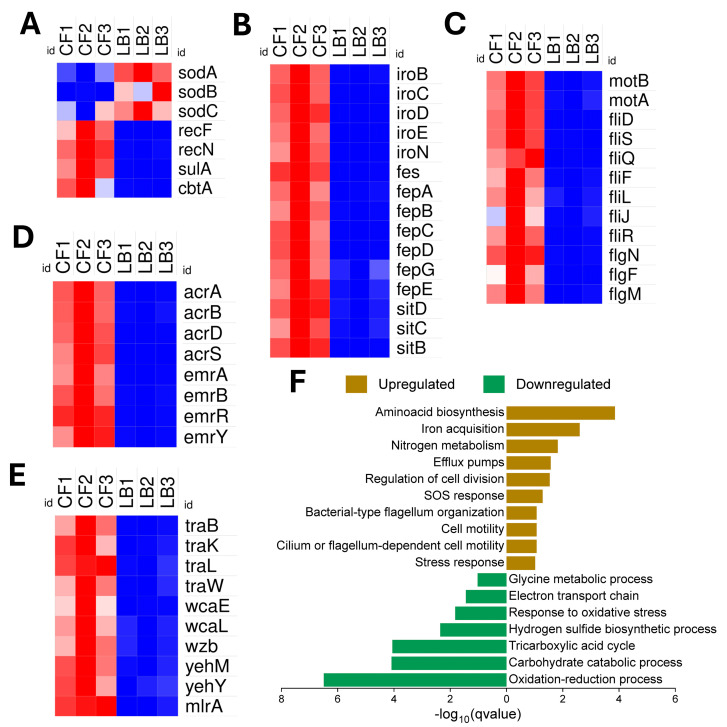
Differential expression and functional enrichment of neonatal meningitis *E. coli* (NMEC) genes under colonic fluid (CF) compared to control Luria Bertani (LB) broth conditions. Heat maps show the differential expression patterns of genes involved in (**A**) oxidative stress response and DNA repair, (**B**) iron acquisition systems, including siderophore biosynthesis and transport, (**C**) flagellar structure and motility, (**D**) efflux pumps, and (**E**) conjugative transfer, biofilm formation, and putative stress-related genes. Differential expression values are presented as normalized log_2_-transformed total counts and color-coded from blue (low expression) to red (high expression). The bar graph depicts the functional enrichment analysis of significantly enriched upregulated (brown bars) and downregulated (green bars) biological pathways based on −log_10_(q-value) (**F**).

In CF, NMEC RS218 demonstrated significant metabolic reprogramming by downregulating energy-intensive pathways, including carbohydrate metabolism, TCA cycle, oxidative phosphorylation, electron transport, hydrogen sulfide synthesis (e.g., *cysD*, *cysN*, *cysH*, *cysI*), glycogen metabolism (*glgP*, *glgC*, *glgA*), and oxidative stress responses (*sodA*, *sodB*, *sodC*) (Figure 4A). Conversely, it upregulated genes essential for survival, such as iron acquisition (*iro*, *fep*, *sit* operons) (Figure 4B), motility (*motA*, *motB*, *fli*, *flg*) (Figure 4C), nitrogen metabolism (*nir*, *gln*), amino acid biosynthesis (*gdhA*, *argB*, *hisG*, *metJ*), efflux pumps (*emr*, *acr*, *mdt*) (Figure 4D), SOS response and DNA repair (*recA*, *recF*, *recN*), cell division regulation (*sulA*, *cbtA*) (Figure 4A), and metal ion homeostasis (*bfr*, *chaA*, *fhuF*). Notably, in CF, NMEC expressed genes involved in bacterial conjugation (*traK*, *traL*, *traW*), biofilm formation (*yehM*, *yehY*, *wca*, *wzb*, *mlrA*) (Figure 4E), and stress response/transport functions (*yajR*, *ybhJ*, *ybiX*, *yedA*), highlighting adaptations crucial for colonization and persistence in the colonic niche. The pathways exhibiting significant upregulation or downregulation are shown in Figure 4F.

### 3.3. Overcoming Nutrient Limitations in the Serum

**Figure 5:**
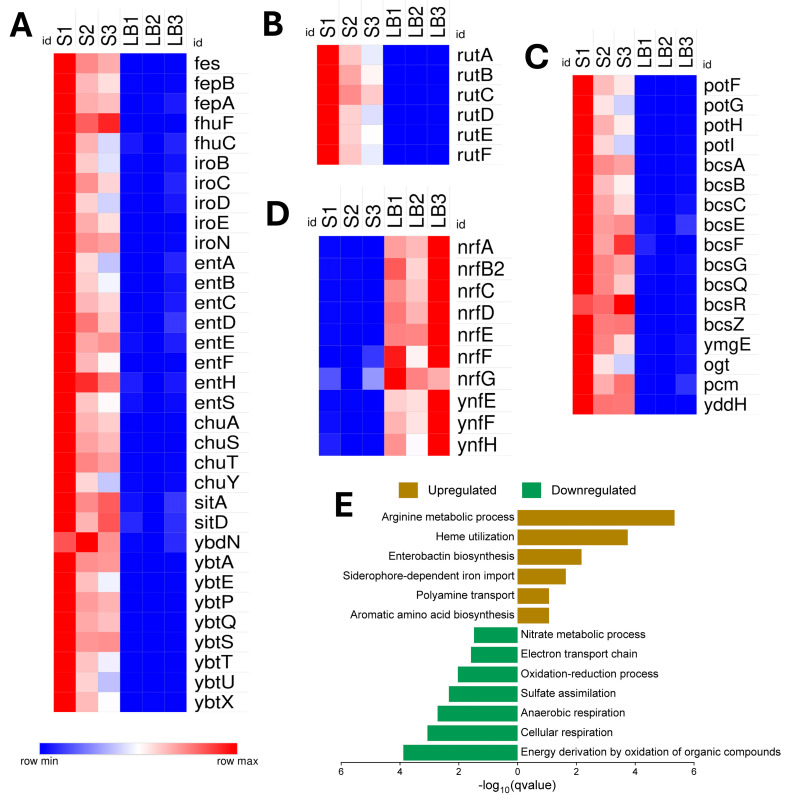
Differential expression and functional enrichment of neonatal meningitis *E. coli* (NMEC) genes in human serum (S) compared to control Luria Bertani (LB) broth. Heat maps showing differential gene expression profiles of genes involved in (**A**) siderophore biosynthesis, enterobactin biosynthesis, iron transport, and acquisition, (**B**) pyrimidine (uracil) catabolism, (**C**) polyamine transport, biofilm formation, and other stress-associated genes, and (**D**) anaerobic respiration. Differential expression values are presented as normalized log_2_-transformed total counts, color-coded from blue (low expression) to red (high expression). The bar graph depicts significantly enriched biological processes for upregulated genes (brown) and downregulated genes (green), plotted by −log_10_(q-value) (**E**).

In serum, where iron is restricted, NMEC RS218 markedly upregulated genes essential for iron acquisition, including siderophore-mediated iron import (*fes*, *fep*, *fhu*, *iro*, *ybd*, *ybt*, *sit*), enterobactin biosynthesis (*ent*), and heme utilization (*chu*, *ddp*) (Figure 5A). Additionally, genes involved in arginine metabolism (*arg* operon, *ast*, *carA*, *carB*), amino acid biosynthesis (*glnA*, *glnH*, *trp*, *aro*), uracil catabolism (*rut*) (Figure 5B), and polyamine transport (*potF*, *potG*, *ydcS*) (Figure 5C) were upregulated, indicating increased nutrient scavenging and metabolic homeostasis. The unique activation of sugar transport (*agaB*, *bglG*, *malK*), biofilm formation (*bcs*, *ymgE*), and stress response/repair mechanism (*ogt*, *pcm*, *yddH*) genes (Figure 5C) underscored the adaptive resilience of NMEC in serum. Conversely, NMEC reduced the expression of genes associated with energy-intensive pathways, including aerobic respiration, oxidative phosphorylation (*fldB*, *cyo*), anaerobic respiration (*nrf*, *ynf)* (Figure 5D), sulfur/nitrogen metabolism (*cys*, *nar*, *nir*), the TCA cycle (*glcB*, *fumC*), and oxidative stress response (*katE*, *katG*), reflecting metabolic adaptation to serum nutrient constraints. Figure 5E highlights the significantly upregulated and downregulated biological pathways of NMEC when grown in serum.

### 3.4. Gene Expression Associated with Blood–Brain Barrier (BBB) Interaction in Human Brain Endothelial Cells (HBECs)

**Figure 6:**
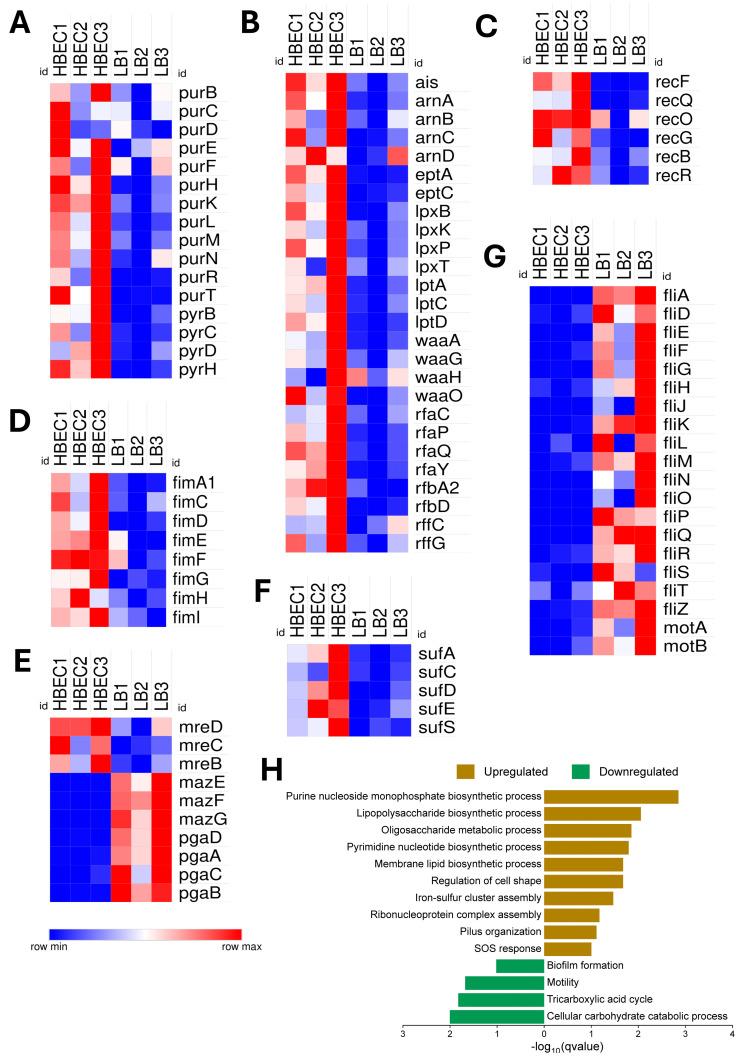
Differential expression and functional enrichment of neonatal meningitis *E. coli* (NMEC) genes in human brain endothelial cells (HBECs) compared to the control Luria Bertani (LB) broth. Heat maps show differential gene expression patterns of genes involved in (**A**) purine and pyrimidine biosynthesis, (**B**) lipopolysaccharide (LPS) modification, transport, and membrane integrity, (**C**) DNA repair, (**D**) adhesion, (**E**) cell shape regulation, toxin–antitoxin systems, and biofilm synthesis, (**F**) iron–sulfur cluster assembly, and (**G**) flagellar biosynthesis and motility. Differential expression values are presented as normalized log_2_-transformed total counts and color-coded from blue (low expression) to red (high expression). The bar graph depicts the significantly enriched biological processes for upregulated genes (brown) and downregulated genes (green), plotted by −log_10_(q-value) (**H**).

NMEC RS218 demonstrated transcriptional adaptation to HBECs by prioritizing rapid proliferation through enhanced nucleoside/nucleotide metabolism (*pur* and *pyr* genes) (Figure 6A) and elevated ribosomal assembly (*rps* genes) for protein synthesis. The increased expression of genes related to lipopolysaccharide (LPS) modification and membrane integrity (*ais*, *arn*, *ept*, *lpx*, *lpt*, *waa*, *rfa*, *rfb*, *rff*) (Figure 6B), SOS response and DNA repair (*rec* genes) (Figure 6C), pilus organization (*fim*) (Figure 6D), cell shape regulation (*mre*) (Figure 6E), and iron–sulfur cluster assembly (*suf*) (Figure 6F) might support NMEC’s endothelial invasion and survival. The unique expression of genes such as *yjeT*, *yjeO* (hypothetical membrane-associated proteins), *wzyE* (polysaccharide biosynthesis), *ppdC* (putative adhesin), and *tssA2* (type V1 secretion system component) suggests additional pathways of NMEC involved in host modulation. Conversely, NMEC downregulated genes associated with carbohydrate metabolism (*ara*, *man*, *gal*), TCA cycle enzymes (*sdh*, *fum*, *icd*), biofilm formation (*maz*, *pga*) (Figure 5E), and extensive motility-associated proteins (*fil*, *mot*) (Figure 6G), reflecting reduced reliance on oxidative pathways and motility during brain endothelial colonization. The enriched pathways of NMEC exhibiting significant upregulation or downregulation in HBEC are illustrated in Figure 6H.

### 3.5. Adapting to the Cerebrospinal Fluid (CSF)

**Figure 7:**
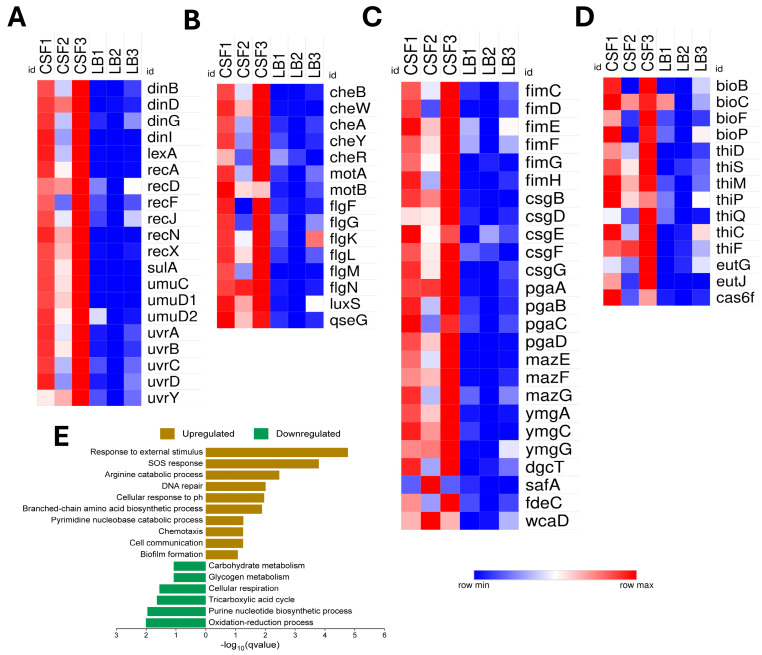
Differential expression and functional enrichment of neonatal meningitis *E. coli* (NMEC) genes in the cerebrospinal fluid (CSF) compared to the control Luria–Bertani (LB) broth. Heat maps showing differential gene expression patterns of genes involved in (**A**) SOS response and DNA repair, (**B**) flagellar motility, chemotaxis, and quorum sensing, (**C**) adhesion and biofilm formation, and (**D**) biotin and thiamine biosynthesis, ethanolamine utilization, and CRISPR-associated Cas. Differential expression values are presented as normalized log_2_-transformed total counts and color-coded from blue (low expression) to red (high expression). The bar graph depicts the significantly enriched biological processes for upregulated genes (brown) and downregulated genes (green), plotted by −log_10_(q-value) (**E**).

NMEC RS218 exhibited distinct transcriptional responses in CSF. Genes linked to the stress-induced SOS response (*dinB*, *dinD*, *dinG*, *lexA*, *recA*, *recN*, *sulA*, *umuC*, *uvrA*, *uvrB*) (Figure 7A), chemotaxis and motility (*cheA*, *cheB*, *cheR*, *cheW*, *cheY*, *motA*, *motB*, *flg*, *fli*), quorum sensing (*luxS*, *qseG*) (Figure 7B), biofilm formation and adhesion (*fim*, *csg*, *pga*, *maz*, *ymgC*, *dgcT*, *safA*, *fdeC*), and colanic acid capsule biosynthesis (*wcaD*) (Figure 7C) were notably upregulated. Additionally, NMEC enhanced amino acid biosynthesis pathways, including methionine (*met* genes), branched-chain amino acids (*ilv* and *leu* genes), aromatic amino acids (*pheA*, *trpB/E*, *tyrA*), activated arginine catabolism (*ast* genes), and uracil metabolism (*rut* genes). Unique enrichment of oxidative stress resistance and membrane integrity (*aphF*, *yciO*, *dsbB*, *ybhP*), biotin and thiamine synthesis (*bioB*, *thiF/S*), ethanolamine utilization (*eutG/J*) (Figure 7D), and CRISPR-associated RNA processing (*cas6f*) might provide a survival advantage to NMEC in this unique niche. Conversely, NMEC downregulated energy-intensive pathways, such as oxidative phosphorylation (*cyd* genes), cellular respiration (*fdh*, *fdn*, *fdo*, *nuo* operons), TCA cycle components (*sucA*, *sdhA-E*, *fumB-D* genes), carbohydrate catabolism (*gal*, *ara*, *fuc* genes), glycogen metabolism (*glg*, *mal* genes), and sugar phosphotransferase systems (*srl*, *gat* operons), indicating a metabolic shift toward conserving energy in the nutrient-limited CSF environment. The enriched pathways of NMEC exhibiting significant upregulation or downregulation in CSF are illustrated in Figure 7E.

### 3.6. Comparative Virulence Strategies into Niche-Specific Adaptations

**Figure 8:**
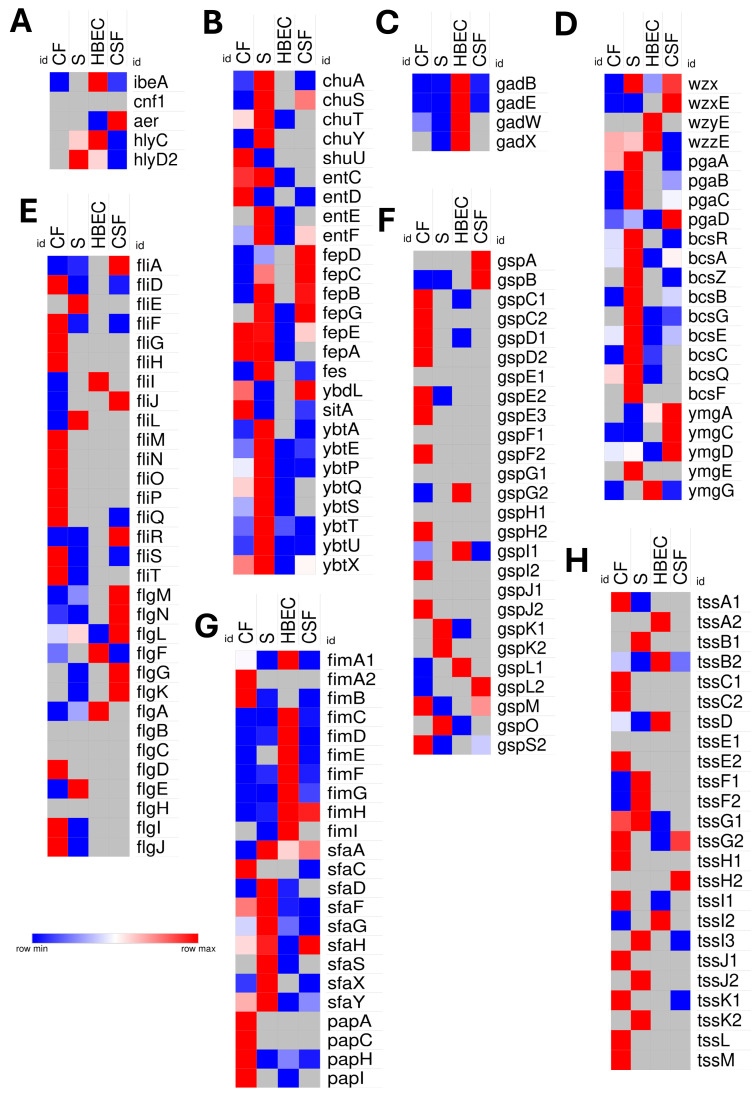
Comparative expression profile of virulence-associated genes of neonatal meningitis *E. coli* (NMEC) under four host-mimicking conditions, namely (CF), serum (S), human brain endothelial cells (HBECs), and cerebrospinal fluid (CSF). Heat maps showing differential gene expression patterns of (**A**) invasion (*ibeA*), cytotoxic necrotizing factor (*cnf1*), aerobactin transport (*aer*), and hemolysin (*hlyD*) genes, (**B**) genes related to iron acquisition systems, (**C**) acid resistance-related genes, (**D**) biofilm- and capsule-associated genes; (**E**) flagellar assembly genes, (**F**) type II secretion system (T2SS)-related genes, (**G**) fimbrial adhesins, and (**H**) Type VI secretion system (T6SS)-associated genes. Differential expression is presented as normalized, log_2_-fold change values, and color-coded from blue (low expression) to red (high expression). Gray tiles indicate absence or undetectable expression.

NMEC RS218 exhibited different transcriptomic profiles across host niches. Notably, *ibeA*, a gene crucial for BBB invasion, was highly upregulated in HBECs, whereas *cnf1* was downregulated in all niches. The aerotaxis gene *aer* showed significant enrichment exclusively in the CSF. Iron acquisition genes related to heme uptake (*chuA*, *chuS*, *chuT*, *chuY*, *shuU*) and enterobactin biosynthesis (*entC*, *entD*, *entE*, *entF*) were upregulated in CF and serum, selectively expressed in CSF, and absent or reduced in HBECs. Siderophore-mediated iron acquisition genes (*fes*, *fep*, *iro*, *ybdL*, *sitA*) also showed niche-dependent expression, whereas *tonB* upregulation was limited to CF and serum. Siderophore biosynthesis genes (*ybt* operon) were strongly activated in the serum but were reduced in HBECs and CSF. Acid-resistance (GDAR) genes (*gadB/E/W/X*) were prominently expressed in HBECs, also found in serum and CSF, suggesting niche-specific acid tolerance in NMEC. Hemolysin genes (*hlyC/D*) associated with cytotoxicity were upregulated in the serum, HBECs, and CSF. Genes responsible for enterobacterial O-antigen gene synthesis and transport (*wzx*, *wzxE*, *wzyE*, *wzzE*) were upregulated in serum, HBECs, and CSF as compared with CF, which might enhance the structural integrity, immune evasion, and BBB traversal of bacteria. Type VI secretion system (*tss* genes) components were differentially expressed in all niches; however, most of them were expressed in CF, emphasizing niche-dependent virulence strategies. Type II secretion system genes (*gsp*) exhibited prominent expression, primarily in CF. Flagellar assembly and motility genes (*fli*, *flg*) were extensively upregulated in CF, followed by serum and CSF, but were minimally upregulated in HBECs. Adhesion factor genes, including type 1 fimbriae (*fimA–I*), S-fimbriae (*sfa*), and P fimbriae (*papH*), demonstrated niche-specific prominence, suggesting tailored adherence strategies. Biofilm-associated genes (*pgaA–D*, *bcs*) were highly expressed in CF, serum, and CSF but were markedly downregulated or absent in HBEC. In contrast, single-species biofilm genes (*ymgA–G*) were notably elevated in the CSF, highlighting the unique biofilm adaptation mechanisms of NMEC in this unique microenvironment (Figure 8).

### 3.7. qRT-PCR Validation of Key Transcriptional Changes

**Figure 9:**
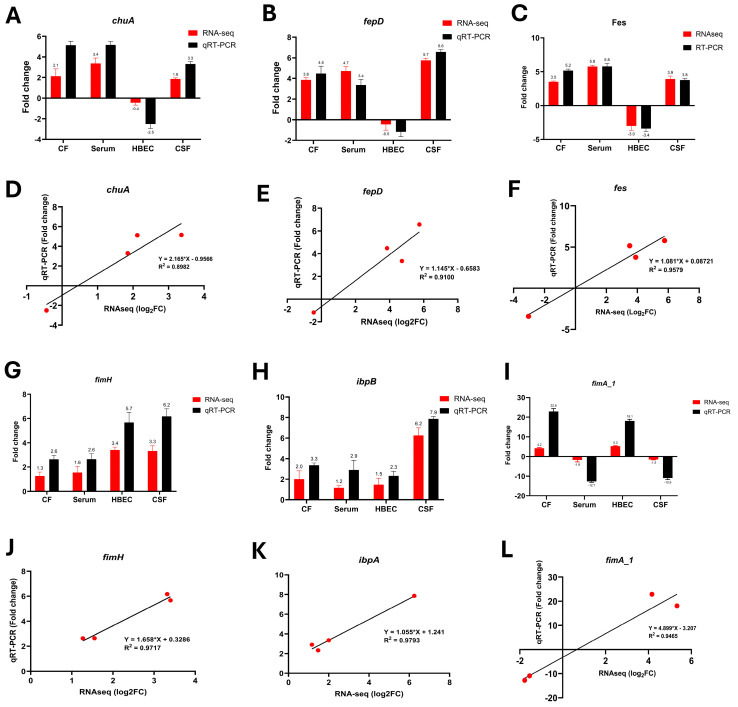
Comparison of RNA-seq and qRT-PCR expression levels of six genes (**A**) *chuA*, (**B**) *fepD*, (**C**) *fes*, (**G**) *fimH*, (**H**) *ibpB*, and (**I**) *fimA1*, across different experimental conditions such as colonic fluid (CF), serum (S), human brain endothelial cells (HBECs), and cerebrospinal fluid (CSF). Red bars indicate RNA-seq log_2_ fold change (log_2_FC) and black bars represent qRT-PCR fold change values. Data are presented as the mean ± standard deviation (SD), and comparisons were made using unpaired *t*-tests with *p* < 0.05 considered statistically significant. RNA-seq log_2_ fold change (X-axis) is plotted against qRT-PCR fold-change values (Y-axis) for (**D**) *chuA*, (**E**) *fepD*, (**F**) *fes*, (**J**) *fimH*, (**K**) *ibpA*, and (**L**) *fimA1*. A strong positive correlation was observed between both datasets, supporting the consistency and reliability of the RNA-seq results. The correlation coefficient (R^2^) and linear regression equations were provided for each gene.

To confirm the observed expression patterns from RNA-seq, the selected transcripts were validated by a qRT-PCR analysis of six selected genes. As shown in Figure 9, the relative transcript levels measured using qRT-PCR were consistent with the corresponding RNA-seq data. Correlation between RNA-seq and qRT-PCR was performed using linear regression analysis (Pearson analysis). A significant positive correlation between the log2 fold change values of RNA-seq values and fold change values of qRT-PCR confirmed the consistency and reproducibility of the RNA-seq analysis.

## 4. Discussion

NMEC is a pathogen with extraordinary resilience, navigating a complex journey from the gut to the bloodstream, and ultimately breaching the CNS. Our transcriptomic analysis of the NMEC strain RS218 across four host-relevant environments—CF, serum, HBECs, and CSF—provides an unprecedented view of how this pathogen fine-tunes its genetic arsenal to exploit each niche. These findings reveal a strategic orchestration of virulence, metabolism, stress resistance, and immune evasion, enabling NMEC to transition from a gut commensal to a deadly neuroinvasive pathogen.

### 4.1. Adaptive Strategies of NMEC for Survival and Persistence in the Colonic Environment

The colon serves as the NMEC’s starting battleground, a densely populated microbial ecosystem in which survival is dictated by resource competition and stress resistance [14]. NMEC displayed a striking metabolic shift, downregulating energy-intensive pathways such as oxidative phosphorylation and the TCA cycle, favoring anaerobic survival in this hypoxic niche, a strategy employed by multiple intestinal and facultative anaerobic pathogens [15]. This shift likely reflects the need to conserve energy under the predominantly anaerobic conditions of the colon while maximizing fitness for competition. Simultaneously, NMEC activated essential survival and nutrient acquisition pathways, prominently upregulating siderophore-mediated iron scavenging systems to counteract host-imposed iron limitation. Additionally, genes involved in anaerobic nitrogen metabolism were elevated, perhaps to ensure sustained energy production under oxygen-deprived conditions [16]. Reactive oxygen species (ROS) production is markedly reduced in the colonic niche, diminishing the need for robust oxidative stress defenses. Consequently, NMEC’s downregulation of *sodA*, *sodB*, and *sodC* likely reflects an adaptive response to conserve energy by minimizing the synthesis of these superoxide dismutases when oxidative threats are minimal [17]. *E. coli* can produce hydrogen sulfide (H_2_S) through cysteine catabolism, but in the colon, where H_2_S is already abundant from other gut microbes, excessive H_2_S production may be unnecessary or even detrimental. By restricting its own H_2_S biosynthesis, NMEC might avoid compounding toxic sulfide levels and save energy. In addition to the downregulation of H_2_S biosynthesis, we also noted the downregulation of genes involved in glycogen metabolism (both synthesis and breakdown), suggesting that NMEC does not invest in carbon storage polymers during colonization.

Iron acquisition is essential for NMEC survival and virulence, particularly in iron-limited colonic environments. NMEC upregulated key siderophore systems, including salmochelin (*iroB*, *iroC*, *iroD*, *iroE*, *iroN*) and enterobactin (*fepA*, *fepB*, *fepC*, *fepD*, *fepE*), to enhance iron uptake. While enterobactin is highly efficient, it is susceptible to host sequestration by lipocalin-2; however, salmochelin evades this defense, giving NMEC a survival advantage. Additionally, the upregulation of the sit operon (*sitA*, *sitB*, *sitC*, *sitD*) supports iron and manganese transport. These strategies, often plasmid-encoded (pRS218), ensure iron sufficiency, priming NMEC for invasion or systemic spread by replenishing bacteria with essential nutrients such as iron [18]. NMEC upregulated nitrate/nitrite reductases in the colonic niche, enabling anaerobic respiration. This mirrors enteric pathogens, such as enterohemorrhagic *Escherichia coli* (EHEC) and *Salmonella*, which utilize host-derived nitrate for growth in the inflamed gut. In addition, the increased expression of ammonium transporters and amino acid deaminases suggests active nitrogen uptake and assimilation, providing sufficient nitrogen for biosynthetic pathways despite competition in the microbiota-rich environment.

NMEC combats intestinal stress by upregulating efflux pumps, such as AcrAB-TolC, a key defense against bile salts, fatty acids, and antimicrobial peptides. This system expels toxic compounds and shields membranes and DNA from bile-induced damage. Increased *acrAB* expression enhances *E. coli* survival by reinforcing the resistance strategy [19]. NMEC upregulated SOS regulators and DNA repair genes, highlighting its response to genotoxic stress in the gut. A functional SOS system aids stable colonization, as *E. coli* mutants, unable to mount an SOS, were outcompeted [20]. This stress may also help NMEC with extra-intestinal invasion. The upregulation of *sulA*, a key SOS-induced cell division inhibitor, in the colonic niche, suggests that NMEC halts cell division to allow DNA repair [21]. This may lead to the bacteria adopting a filamentous state in the gut while resisting phagocytosis. A similar mechanism has been reported in UPEC, in which the filaments help to evade neutrophil engulfment in UTIs [22]. NMEC may utilize this strategy in the gut or during translocation to extraintestinal niches. Unlike most *E. coli*, which reduce motility in the gut [23], NMEC upregulated flagellar and chemotaxis genes (*fli*, *flg*, and *mot*), suggesting active movement. This likely aids in navigating NMEC through the gut to locate epithelial sites for adherence and optimize nutrient accessibility while enhancing their persistence and potential for dissemination.

A striking finding of the NMEC transcriptome in CF was the upregulation of plasmid transfer (*tra*) genes. The IncFIB/IIA virulence plasmid (pRS218) in NMEC RS218 carries a full *tra* operon [18], suggesting an active horizontal gene transfer in the dense gut microbiome. This process is enhanced in the gut’s dense microbial environment, where biofilm formation supports cell-to-cell contact [24]. Concurrently, NMEC upregulated genes involved in exopolysaccharide production, including curli fimbriae and colanic acid (*wca*), which promote biofilm formation in the gut mucosa. This biofilm lifestyle might shield NMEC from bile, host immunity, and mechanical shear forces.

### 4.2. NMEC’s Metabolic Adaptations to Overcome Nutrient Limitations in the Bloodstream

Pathogens invading the bloodstream face “nutritional immunity,” where the host tightly sequesters essential nutrients like iron to impede microbial growth [25]. The serum contains virtually no free iron due to iron-binding proteins (e.g., transferrin and lactoferrin), making iron acquisition a critical survival strategy for bacteria [26]. NMEC responds by massively upregulating iron-scavenging systems to overcome this iron limitation. Notably, NMEC induces multiple siderophore-mediated uptake pathways, such as those in the colon, that chelate and import iron from host sources. In addition, NMEC upregulated the *chu* operon for heme uptake, enabling iron extraction from hemoglobin. ChuA binds to hemoglobin-heme complexes, while ChuS facilitates heme transport and degradation [27]. Induced in serum, this system allows NMEC to scavenge iron during invasive infection, especially when hemolysis or tissue damage releases host hemoglobin [28]. By upregulating the heme iron and siderophore systems, NMEC ensures that no source of iron is left untapped.

Beyond iron, the blood serum is generally a nutrient-poor milieu for bacteria, lacking many free sugars and amino acids that are abundant in the gut. To adapt, NMEC rewires its metabolism, upregulates pathways for alternative nutrient utilization, and de novo synthesizes key metabolites such as arginine and aromatic amino acids. Another notable adaptation is the upregulation of the rut operon (*rutABCDEF*), which facilitates the catabolism of uracil. This pathway enables NMEC to utilize uracil as a nitrogen source [29], thereby supporting growth and persistence in serum and systemic infection. Additionally, NMEC upregulated polyamine transport genes including *potF*, *potG*, and *ydcS*. Polyamines, such as putrescine and spermidine are vital for cellular functions, and their uptake is crucial for maintaining cellular homeostasis, particularly under stressful conditions [30].

Concomitant with the activation of nutrient scavenging and stress survival mechanisms, NMEC downregulated energy-intensive pathways, as evidenced by the decreased expression of genes involved in the electron transport chain, anaerobic respiration, sulfate assimilation, nitrate metabolism, the TCA cycle, and oxidation–reduction processes. This strategic metabolic shift allows NMEC to conserve resources and adapt to nutrient constraints in the serum environment. NMEC also upregulated genes associated with sugar transport and metabolism, stress response and repair mechanisms, biofilm formation (*bcsF*, *ymgE*), and other transport-related functions. This coordinated gene expression underscores NMEC’s ability to adapt to its metabolism, enhance stress resilience, and establish persistent infections in the host bloodstream.

### 4.3. Metabolic and Virulence Adaptations of NMEC During Blood–Brain Barrier Invasion

NMEC displayed robust induction of purine (*pur*) and pyrimidine (*pyr*) biosynthetic genes upon interacting with HBECs. Earlier findings showed that *purA* (adenylosuccinate synthase) was preferentially expressed when NMEC associated with brain endothelium, and multiple *pyr* operon genes (*pyrB*, *pyrC*, *pyrD*, etc.) showed ~2-fold higher expression in bacteria bound to HBECs [31]. This upregulation suggests that NMEC accelerate nucleotide production to fuel DNA replication and RNA synthesis in the nutrient-poor BBB microenvironment. In addition to nucleotide biosynthesis, NMEC enhanced their protein production machinery to support survival and growth in HBECs. Transcriptional data indicated an increased expression of ribosomal assembly genes (*rps*) in HBEC-associated NMEC. This reflects the need for more ribosomes to elevate protein synthesis, enabling the bacterium to quickly produce the stress response proteins and virulence factors required at the BBB.

NMEC adapts to the BBB by modifying its LPS structure to reinforce membrane integrity and resist host defenses. Transcriptomic data revealed increased expression of the L-Ara4N (*arn* locus) and PEtN (*ept* locus) enzymes, which modify the lipid A portion of LPS. These modifications reduce the negative charge on the bacterial surface, repelling cationic antimicrobial peptides (CAMPs) of the host [32]. By neutralizing the charge of lipid A, NMEC decreases the affinity of host defensins and antimicrobial peptides for their outer membrane, enhancing survival against innate immune attacks. NMEC also upregulated genes for LPS biosynthesis, LPS core assembly, and LPS transport to the outer membrane. This LPS remodeling may act as a fortified barrier, aiding NMEC persistence during BBB infiltration. Like the serum, the brain endothelium is highly iron-restricted owing to host nutritional immunity. To counter this, NMEC upregulated iron-scavenging systems, notably *suf* genes, which encode an alternative Fe-S cluster assembly pathway. Under iron starvation or oxidative stress conditions, *E. coli* shifts from the housekeeping Isc system to Suf, ensuring essential Fe-S enzyme biogenesis. In NMEC, *suf* induction likely serves two roles: (1) maximizing scarce iron utilization and (2) repairing Fe-S clusters damaged by host reactive oxygen species [33]. This mirrors the strategy of UPEC in the iron-limited urinary tract, thereby aiding the adaptability of NMEC to hostile environments [25].

At the BBB, NMEC faces intense oxidative and genotoxic stresses from immune-derived ROS, RNS, and antimicrobial compounds. Hence, NMEC upregulation of SOS DNA repair genes (*rec*) is likely to counteract the DNA damage inflicted by macrophages and microglial oxidative bursts. This rapid repair preserves the bacterial viability during CNS invasion. Such an adaptation aligns with a key survival strategy for successful pathogens by activating DNA repair and stress tolerance mechanisms to withstand immune-generated genotoxic stress [34]. NMEC strongly upregulated pilus/fimbrial gene clusters, particularly type 1 fimbriae (*fim*) and P pili (*pap*), implying a need to enhance adhesion to HBECs. Type 1 fimbriae, a key virulence factor in *E. coli* K1 meningitis, are predominantly in the “phase-on” state during endothelial interaction, with *fimH* deletion drastically reducing NMEC binding and invasion [35]. P pili, typically linked to UPEC kidney infections, also show increased expression in NMEC, suggesting a complementary adhesion mechanism. Their ability to recognize Galα(1-4)Gal moieties may facilitate the binding of NMEC to brain endothelial glycoproteins. This multi-fimbrial strategy likely strengthens attachment, increases avidity, and helps NMEC withstand shear forces in the bloodstream.

Beyond well-known virulence factors, NMEC upregulate unique or less-characterized genes during BBB transit, including *wzyE*, *tssA2*, and *ppdC*. The *wzyE* encodes the polymerase for the enterobacterial common antigen (ECA), a conserved outer membrane polysaccharide. By polymerizing ECA’s trisaccharide units of ECA into long chains, NMEC may enhance outer membrane stability and immune evasion during brain endothelial interaction. While ECA’s precise function remains unclear, a thicker or more abundant ECA layer could shield NMEC from complement attack, modulate immune recognition, or act as a decoy for antibodies, aiding its persistence at the BBB. The *tssA2* gene, part of T6SS, was notably upregulated in NMEC, suggesting T6SS activation during BBB traversal. T6SS, a phage-like contractile system, primarily aids interbacterial competition but can also deliver effectors into host cells [36]. *tssA* is essential for T6SS assembly and firing [37]. Its induction implies that NMEC may use T6SS to modulate the immune response or alter the endothelium, facilitating bacterial passage [38]. The *ppdC*, which encodes a part of the cryptic type IV pilus or T2SS, is typically low in *E. coli* K-12 [39], but is upregulated in NMEC during HBEC interaction. The HBEC-specific upregulation of *ppdC* suggests that NMEC may turn on this usually silent machinery in response to host signals. A functional type IV pilus may enhance adhesion, twitching motility, or microcolony formation on the endothelium, similar to *Neisseria* and *Pseudomonas* [39].

As a common strategy among bacterial pathogens to encounter nutrient-restricted or hypoxic conditions, NMEC downregulated aerobic respiration in the BBB microenvironment. Additionally, limiting oxidative metabolism could help NMEC evade immune detection, as ROS generation is often linked to aerobic respiration. The ability to dynamically adjust metabolism likely enhances NMEC persistence and invasion at the BBB, facilitating CNS entry. Concomitant with metabolic remodeling, NMEC suppressed genes involved in biofilm formation (*maz* and *pga*) and motility (*fil* and *mot*), aligning with a sessile, host-adapted phenotype. By downregulating these genes, NMEC may sacrifice the formation of thick biofilms on the brain endothelium. A biofilm could impede movement across the BBB and trigger strong immune responses; thus, NMEC likely remains in a more diffuse, planktonic, or microcolony state conducive to translocation. Likewise, motility genes (flagellar proteins and chemotaxis regulators) are turned off or attenuated once NMEC attach to HBECs. For example, flagellin (*fliC*) expression was significantly repressed in endothelial-bound *E. coli* K1 [31], even though the flagellar machinery may have been crucial for initially reaching the BBB. The downregulation of motility-related genes upon contact is a common strategy in UPEC and other extraintestinal pathogenic *E. coli* (ExPEC) flagella when adhering to host cells to avoid triggering immune detection, as flagellin is highly immunogenic in addition to contributing to stabilizing bacterial attachment to host cells [25].

### 4.4. Survival and Persistence Strategies of NMEC in Cerebrospinal Fluid

Once inside the CSF, NMEC faces intense oxidative and immune stress that can damage DNA. In response, NMEC dramatically upregulates SOS regulon genes (e.g., *recA*, *lexA*, *dinB*), central to DNA repair and mutagenesis [40]. The activation of *recA* triggers the autocleavage of the LexA repressor, unleashing over 50 DNA repair genes that fix lesions or bypass them with error-prone polymerases [41]. This SOS response is crucial for bacterial persistence under stress; it repairs oxidative DNA damage (such as that from neutrophil-derived H_2_O_2_). It increases mutation rates, fueling adaptation and survival in the hostile CSF milieu. Notably, SOS induction is linked to the formation of persister cells and biofilms in *E. coli*, probably giving NMEC a further survival advantage during prolonged CSF infection [41]. In contrast to the flagellar and chemotaxis gene downregulation observed in HBECs, NMEC showed increased expression of chemotaxis and flagellar motility genes (e.g., *che*, *mot*, *flag*, and *fli*) in CSF. Flagella-driven motility likely helps NMEC disseminate through the CSF, reach target surfaces (meningeal or ependymal cells) for colonization, and guide the bacteria toward nutrient gradients or protective niches. In response to immune clearance, NMEC appears to enhance the expression of biofilm-promoting and adhesion genes (e.g., *fim* for type-1 fimbriae, *csg* for curli fibers, *pga* for poly-β-1,6-N-acetylglucosamine, *yeh* fimbrial operon, and regulators such as *ariR*). Collectively, these factors facilitate the formation of microcolonies or biofilms within the CSF niche. NMEC likely uses these structures to stick to ependymal or meningeal surfaces, forming sessile communities that are harder for immune cells to eradicate. The *pgaABCD* locus, which synthesizes the polysaccharide, Poly-β-(1–6)-N-acetylglucosamine (PNAG), is also upregulated–this locus is essential for intercellular adhesion and biofilm matrix in *E. coli* [42].

NMEC upregulated quorum sensing (QS) genes in the CSF, including *ariR* (*yliH/bssR*), which modulates biofilm formation and stress tolerance, and *luxS* driving AI-2 signaling for coordinated gene expression [43]. AI-2 influences motility and biofilm formation [44], suggesting that NMECs adapt their behavior based on population density. Additionally, NMEC induced *qseG*, a QS regulator linked to the adrenergic signaling cascade in EHEC, suggesting host-derived signal sensing [44]. This may regulate motility, biofilm formation, and virulence gene expression in response to host stress hormones. NMEC upregulated amino acid biosynthesis to survive nutrient-poor CSF, particularly for methionine, branched-chain amino acids (*ilv*/*leu* operons), and aromatic amino acids (shikimate pathway). This mirrors other meningitis-causing bacterial pathogens, which increase branched-chain amino acid synthesis to compensate for CSF amino acid scarcity [45]. NMEC becomes nutritionally self-sufficient in the CSF and no longer relies on the host. Concomitant with amino acid biosynthesis, NMEC undergoes a broad metabolic shift in the CSF, characterized by suppressing oxidative phosphorylation, the TCA cycle, and carbohydrate catabolism. This is likely to reflect a strategy to conserve energy and adjust to the substrates available in the CSF.

In CSF, NMEC uniquely upregulated *cas6f*, a gene encoding a Type I-F CRISPR-Cas system component, indicating that this system may have functions beyond phage defense. CRISPR activation may counter prophage induction triggered by host stress, maintain genomic integrity, and limit deleterious mobile elements [46]. Emerging evidence suggests that CRISPR-Cas regulates gene expression and virulence, indicating its role in NMEC’s stress resistance and adaptation during infection [47]. In the CSF, NMEC upregulates ethanolamine utilization (*eut*) genes, allowing NMEC to utilize ethanolamine from lysed host cells in CSF as a nutrient source [48]. Beyond metabolism, EutR, the *eut* regulator, may co-activate virulence genes, such as adhesion and toxin genes [49]. NMEC ethanolamine utilization provides a nutrient source and may also trigger virulence gene expression, linking metabolism to pathogenesis and strategically adapting to the host environment.

Finally, NMEC appears to strengthen its capsular shield beyond the hallmark K1 polysialic acid capsule, a key virulence factor that protects NMEC against phagocytosis and complement attack. Our transcriptomic data revealed the upregulation of *wcaD* in the CSF, suggesting increased colanic acid production, an exopolysaccharide typically induced under stress via the Rcs phosphoregulatory system [50]. The addition of a colanic acid layer atop the K1 capsule may further shield NMEC from immune clearance, making them more resistant to neutrophil and microglial attacks in the CSF. This contrasts with CF, where colanic acid production may aid NMEC in survival and competition within the gut microbiota.

### 4.5. Niche-Specific Virulence Strategies

NMEC exclusively upregulated *ibeA* in HBECs, highlighting its role in BBB invasion. IbeA, an invasin, binds vimentin on brain endothelial cells, triggering signaling that facilitates CNS entry [51]. This niche-specific expression underscores the importance of IbeA in NMEC pathogenesis, promoting tight adhesion and penetration of the BBB during meningitis. However, NMEC downregulated *cnf1* under all the test conditions. While *cnf1* enhances invasion in some NMEC strains [52], it is absent in ~27% of strains [53] and may be silenced by global regulators such as H-NS [54]. This suggests that NMEC prioritizes invasins and capsules over cytotoxins for stealthy BBB entry, avoiding excessive host damage or immune activation. The limited role of CNF1 implies that targeting this toxin may have a minimal therapeutic impact on NMEC infections. In CSF, NMEC upregulated *aer*, an aerotaxis receptor that guides bacteria toward oxygen-rich microenvironments [55]. Given CSF’s low oxygen and nutrients in the CSF, this response will likely help NMEC cluster near blood vessels or meninges, optimizing survival and growth. Aerotaxis activation reflects NMEC’s metabolic flexibility, allowing it to adapt to hypoxic conditions and thrive in the brain.

This study demonstrates that NMEC employs niche-specific iron acquisition strategies across different host environments to overcome iron limitation, a critical factor for bacterial survival and virulence. In the CF and serum, NMEC upregulated heme uptake genes (*chuA*, *chuS*, *chuT*, *chuY*, *shuU*), facilitating the utilization of heme as an iron source. For instance, the ChuA receptor enables direct iron acquisition from heme, as in *Shigella* species [56]. This adaptation allows NMEC to exploit heme from dietary sources or lysed erythrocytes, providing a growth advantage in iron-restricted environments. Conversely, in iron-depleted CSF, NMEC prioritizes siderophore-mediated iron acquisition. The upregulation of genes involved in enterobactin biosynthesis (*entC*, *entD*, *entE*, *entF*, *entH*, *entS*, *ybdL*, *ybdZ*, *ybtA*, *sitA*) and siderophore transport (*fes*, *fepA*, *fepB*, *fepC*, *fepD*, *fepG*, *iroE*, *iroN*) equips NMEC to synthesize and import high affinity siderophores that sequester iron from host proteins. This adaptation is crucial in the CSF, where free iron is scarce, and siderophores like enterobactin become essential for bacterial survival [57]. Notably, these iron acquisition systems were downregulated during interaction with HBECs.

NMEC faces intense oxidative, thermal, and osmotic stress during invasive infection, particularly in the inflamed CNS. *clpS*, an adaptor for the ClpAP protease, was highly expressed in the CSF, suggesting a vigorous response. ClpS helps degrade oxidatively damaged proteins, prevents toxic aggregation, and plays a role in iron homeostasis by regulating iron release under H_2_O_2_ stress [58]. Its upregulation indicates that NMEC actively counteracts oxidative immune defenses in the CNS, thereby enhancing survival. A robust Clp-mediated stress response may promote NMEC’s persistence inside phagocytes or during fever-induced stress [59]. Targeting ClpS/ClpP could be a potential therapeutic approach to weaken NMEC’s ability to withstand host defenses.

NMEC modulates the glutamate-dependent acid resistance (GDAR) system based on the host environment. GDAR genes (*gadB*, *gadE*, and *gadW/X*) were highly expressed in HBECs, CF, and serum but downregulated in CSF, reflecting adaptation to pH stress. The GDAR system, the most potent acid resistance mechanism in *E. coli*, consumes protons by converting glutamate to gamma-aminobutyric acid (GABA), which buffers against acidity [60]. In the gastrointestinal tract, NMEC likely activates the *gad* to withstand stomach acidity (~pH 2) or acidic byproducts of gut fermentation. In blood and HBEC interactions, NMEC may face acidic stress within phagolysosomes (pH 4–5), where GDAR enhances survival against immune defenses. The regulators GadE, GadW, and GadX orchestrate a global acid resistance response, activating stress genes and chaperones to protect against low pH [61]. Interestingly, GDAR genes were not induced in the neutral pH environment in CSF, suggesting that NMEC toggled acid resistance where needed (gut, blood, intracellular) and turned it off in CSF.

NMEC also modulates hemolysin expression (*hlyC/D*) across host microenvironments, suggesting the controlled use of α-hemolysin (HlyA) to balance invasion and immune evasion. HlyA, a pore-forming cytolysin, requires HlyC for activation and HlyD for Type I secretion [62]. In serum and HBECs, the upregulation of *hlyC/D* indicates a potential role for HlyA in immune evasion and host–cell interactions. Hemolysin may help NMEC survive in the blood by killing phagocytes or modulating endothelial cells to facilitate persistence and dissemination [63]. In CSF, *hlyC/D* expression was lower than in serum and HBECs, suggesting a limited role for hemolysin in this niche. NMEC may use hemolysin for nutrient acquisition or immune modulation, but its controlled expression likely prevents excessive inflammation in the brain. In the gut (i.e., CF), *hlyC/D* was not expressed, indicating that NMEC does not rely on hemolysin for intestinal colonization, likely favoring other virulence strategies, such as adhesins and biofilm formation.

The differential expression of the secretion system genes under four test conditions indicated their varying roles across host environments. For example, *tssA2* upregulation in HBEC suggests T6SS-mediated host modulation, aiding cytoskeletal remodeling and tight junction disruption to facilitate BBB penetration [64]. T6SS expression in CF and serum implies its role in immune evasion, bacterial competition, or host interactions. *tssH2* induction in CSF signals during T6SS firing cycles likely targets host phagocytes or microbial competitors. This adaptive T6SS deployment supports the invasion of the BBB and survival in the CNS. Most T2SS components (*gsp* genes) also showed varying expressions across niches. T2SS, a general secretory pathway, exports folded periplasmic proteins such as enzymes and toxins. Its exclusive induction in CF implies that NMEC uses T2SS-secreted factors (e.g., mucinases, proteases, and biofilm components) to penetrate the neonatal intestinal mucus layer, modulate the gut epithelium, or condition the niche for persistence. This mirrors enteric pathogens, such as EHEC, where T2SS enhances adhesion and colonization. Unlike diarrheagenic *E. coli*, NMEC lacks an LEE-encoded T3SS and likely relies on T2SS and adherence fimbriae for initial gut establishment before downregulating T2SS upon systemic spread [65]. This controlled activation conserves energy, ensuring that T2SS is only expressed when needed.

NMECs fine-tune their adhesins and biofilm-related genes across host environments to optimize colonization, invasion, and persistence. F1 fimbriae (*fim* operon) were expressed in all niches but were highly upregulated in HBECs, supporting BBB attachment via mannose-binding FimH. This resembled UPEC’s bladder colonization of UPEC, but NMEC repurposed it for brain endothelial adherence. Expression decreased in the CSF, indicating a reduced need for surface adhesion post-BBB crossing. Similarly, S-Fimbriae (*sfa* operon) was expressed in all niches but was highly upregulated in serum, suggesting a role in bloodstream survival and dissemination. S-fimbriae bind sialylated glycoproteins, helping NMEC evade clearance by adhering to the vascular endothelium or circulating cells. The high expression of *papH*, a subunit of P pili involved in pilus assembly and termination [66], in CF and HBECs indicates a role for P pili in gut colonization and BBB interaction in NMEC. This suggests that NEMC could leverage targeting and blocking PapG adhesin to reduce gut colonization and bloodstream invasion. Biofilm matrix genes (*pga*, *bcs*, *ymg*) were also upregulated in CF, serum, and CSF, suggesting that NMEC adopts a biofilm-like state to resist immune clearance and environmental stress in these niches. In contrast, the downregulation of these genes in HBECs aligns with the transition of NMEC to a planktonic, invasive phenotype, which is better suited for crossing the BBB. This dynamic virulence gene regulation highlights the ability of NMEC to shift between adhesion, invasion, and biofilm formation, ensuring efficient survival and dissemination across varying environments within the host.

While our findings provide important insights into the transcriptomic adaptations of NMEC in host-mimicking environments, there are certain limitations to consider. The study was conducted using in vitro models, which, although useful for controlled analysis, do not fully capture the complexity of in vivo host–pathogen interactions. Furthermore, the absence of in vivo validation restricts the extrapolation of these results to actual disease conditions. Future studies involving appropriate animal models will be necessary to validate these findings and to further elucidate the molecular mechanisms underlying NMEC pathogenesis in vivo.

## 5. Conclusions

This study provided comprehensive insights into NMEC’s sophisticated adaptive strategies across diverse host environments, highlighting its ability to dynamically modulate metabolism, stress responses, adhesion, and virulence factors. NMEC demonstrates remarkable niche-specific gene regulation, optimizing colonization in the gut, persistence in serum, precise invasion at the BBB, and robust survival within the CSF. By selectively activating iron acquisition systems, oxidative stress responses, adhesins, biofilm matrix production, and secretion systems according to environmental demands, NMEC balances its virulence with that of stealth to maximize survival and dissemination. These transcriptomic insights will enhance our understanding of NMEC pathogenesis and reveal potential targets, such as fimbrial adhesins, biofilm regulators, iron acquisition pathways, and stress response adaptors, for developing targeted therapeutic interventions to combat neonatal meningitis.

## Data Availability

The original data presented in the study are openly available in the Gene Expression Omnibus (GEO) database in NCBI under the accession number GSE291265.

## Supplementary Materials

The following supporting information can be downloaded at: https://www.mdpi.com/article/10.3390/pathogens14050485/s1, File S1: Primers used for qRT-PCR analysis; File S2: Differential expression analysis data (unfiltered) of *E. coli* RS218 in colonic fluid (CF), serum (S), human brain endothelial cells (HBECs), and cerebrospinal fluid (CSF) compared with Luria Broth (LB) as control, performed on the CLC Genomics expression browser; Figure S1: Heat map of all differentially expressed genes in colonic fluid (CF); Figure S2: Heat map of all differentially expressed genes in serum (S); Figure S3: Heat map of all differentially expressed genes in human brain endothelial cells (HBECs); Figure S4: Heat map of all differentially expressed genes in cerebrospinal fluid (CSF).

## Abbreviations

The following abbreviations are used in this manuscript:

NMEC	Neonatal Meningitis *Escherichia coli*
CF	Colonic fluid
S	Serum
HBEC	Human brain endothelial cells
CSF	Cerebrospinal fluid
BBB	Blood–brain barrier
CNS	Central nervous system
LB	Luria–Bertani broth
DGE	Differentially expressed genes
TCA	Tricarboxylic acid
CRISPR	Clustered Regularly Interspaced Short Palindromic Repeats
