# Supplementary material for: From the Gut to the Brain: Transcriptomic Insights into Neonatal Meningitis Escherichia coli Across Diverse Host Niches
Source: Pathogens. 2025 May 15;14(5):485. doi: 10.3390/pathogens14050485 (PMC12114223; doi:10.3390/pathogens14050485)
Supplement: Supplementary file 1 [file pathogens-14-00485-s001.zip › Supplementary File S1.pdf]

**Primer sequences used for qRT-PCR**

***chuA:***

Fp_CACCTTGCCAACGTTTGCTT

Rp_GACCATCATAGGCGCTTCGA

***FepD:***

Fp_CGATCAACTGCGTTTCTGGC

Rp_TAAGCACCGGGATTAGCACC

***Fes:***

Fp_TCACCATCAGAACAGCCAGC

Rp_GGCATTGAGTTGTGTCGTCC

***FimH:***

Fp_CCGGTGGCGCTTTATTTGAC

Rp_GTCGCAAAATAAGCACGGCA

***IbpB:***

Fp_AAAATGGCTGCATCAAGGGC

Rp_GTTGCGCCAGAGACTTCCAT

***FimA:***

Fp_GACGGTGCGACATTTAGTGC

Rp_ACCGGTTGCAAAATAACGCG
